# Editorial: Chrononutrition and health

**DOI:** 10.3389/fnut.2024.1516940

**Published:** 2024-11-14

**Authors:** Valentini Konstantinidou, Humaira Jamshed

**Affiliations:** ^1^Medoliali S.L. (Dnanutricoach^®^), Barcelona, Spain; ^2^Faculty of Health Sciences, Universitat Oberta de Catalunya (Open University of Catalonia, UOC), Barcelona, Spain; ^3^Integrated Sciences and Mathematics, Dhanani School of Science and Engineering, Habib University, Karachi, Pakistan

**Keywords:** chronotype, circadian, metabolism, meal timing, personalized nutrition

Chrononutrition is an emerging field that explores how the timing of food intake affects our health and wellbeing. It includes behaviors like intermittent fasting, meal skipping, breakfast or dinner latency, etc. By examining the intersection of nutrition, circadian rhythms, and metabolism, chrononutrition sheds light on how eating patterns can impact not only our weight (1) and metabolic health (2) but also our mental and emotional wellbeing (3). This special edition brings together a collection of articles that advance our understanding of the intricate relationships between what we eat when we eat, and how these choices interact with our biological clocks.

## Setting the context: why timing matters

The idea that “when” we eat matters is grounded in circadian biology—the internal clock system that regulates various physiological processes over a 24-h cycle (4). Disruptions to this clock, such as those caused by irregular meal timing or shift work, have been increasingly linked to metabolic disorders, poor sleep quality, and cardiovascular issues (5). The research contributions in this edition shed some light on the importance of our circadian rhythms in health and disease.

## Contribution to the field

**Figure 1:**
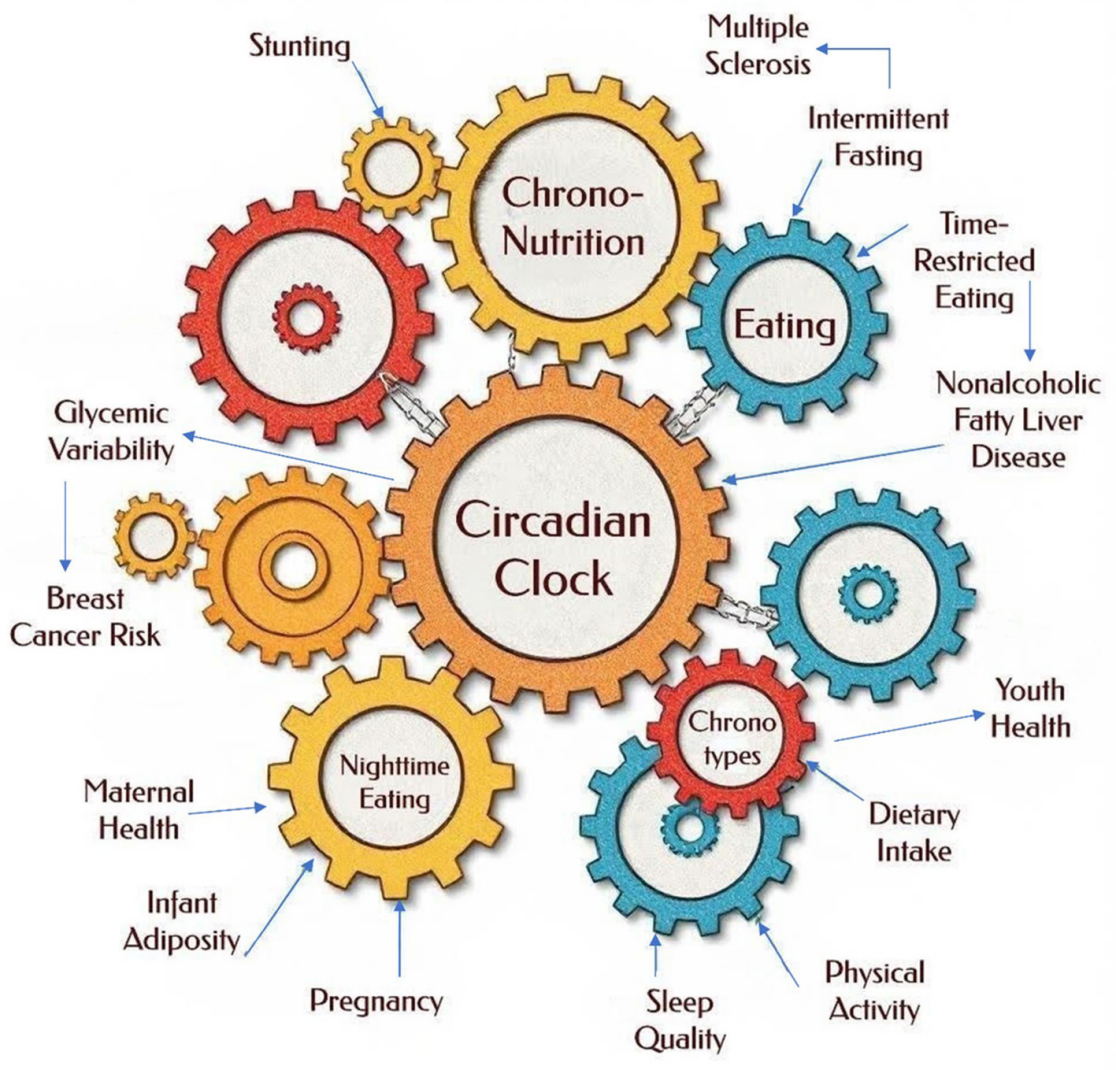
Chrononutrition: the interconnected elements of timing, metabolism, and health.

This Research Topic collected original research and review articles on the latest advances in chrononutrition in human studies from a wide range of disciplines (Figure 1). Fifty-two authors from around the globe participated in these eight studies showing mounting interest in the field.

The first study by Mortaş et al., highlights how chronotype affects dietary habits. Individuals with an evening chronotype were found to be more prone to unhealthy eating habits compared to morning types. These findings suggest that personal chronotype could be a key factor in developing individualized nutritional strategies to improve dietary attitudes and behaviors.

Building on this theme, the exploratory analysis on low-glucose eating by Jospe et al., investigates how meal timing impacts glycemic variability in postmenopausal women. This study found that low-glucose eating (LGE)—a form of timed eating—was associated with improved glycemic control, even without weight changes. This suggests that strategic meal timing could offer significant benefits for managing metabolic risks, particularly in vulnerable populations.

Another cross-sectional study by Günal examines the connections between chronotype, sleep, activity, and diet. The study found that evening chronotypes often face poorer sleep quality and less favorable dietary patterns compared to morning chronotypes. Additionally, active individuals experienced better sleep and improved dietary intake, suggesting the importance of synchronized lifestyle behaviors for optimal health outcomes.

The review article on chrononutrition and stunting by Taslim et al., shifts the focus to maternal and early childhood nutrition. It highlights how maternal chrononutrition—especially during pregnancy and early life—affects stunting in children. Maintaining a balanced diet in harmony with circadian rhythms during critical growth periods could be instrumental in preventing stunting and supporting healthy physical and cognitive development.

The systematic review by Lin, Wang, Huang provides evidence that time-restricted eating (TRE) can be beneficial for managing non-alcoholic fatty liver disease. Improvements in liver health indicators, such as intrahepatic triglyceride levels, were noted across multiple trials. While promising, further research is needed to confirm these benefits and establish TRE as a clinical recommendation for disease management.

In a similar vein, the second systematic review by Lin, Wang, Guo summarizes evidence supporting intermittent fasting as a potential dietary intervention to improve the quality of life for patients suffering from multiple sclerosis. Though preliminary, these findings suggest that meal timing could play an important role in managing this complex neuroimmune disorder.

Lastly, the observational study by Rodríguez-Cano et al. found that night-time eating during pregnancy was associated with increased fat mass in infants at 6 months. This finding suggests that maternal meal timing can have significant long-term effects on offspring health, emphasizing the need for targeted guidance during pregnancy.

## Emerging themes and future directions

Several key themes emerge from the research presented in this special edition. One major takeaway is the critical role of early eating—research suggests that consuming more calories earlier in the day aligns better with our circadian rhythms and supports metabolic health (2). Another recurring theme is the influence of chronotype on dietary habits and sleep quality, indicating that personalized nutrition strategies based on chronotype could yield more effective health outcomes (6).

The potential benefits of specific eating patterns, such as low-glucose eating, time-restricted feeding, and intermittent fasting, are further highlighted, particularly for managing chronic health conditions like NAFLD and MS. Despite these advances, significant gaps remain. More research is needed to determine the long-term impact of chrononutrition interventions on diverse populations, including those who face unique challenges, such as shift workers or individuals with chronic illnesses.

Additionally, the role of chrononutrition in maternal and infant health represents a promising but underexplored area that warrants further investigation. Future studies could focus on how chrononutrition strategies can optimize health outcomes across generations, beginning *in utero* and continuing through early childhood development.

## Broader implications

The findings presented here carry significant implications for public health and clinical practice. We are optimistic that these contributions have the potential to inspire future research and facilitate the integration of chrononutrition principles into clinical practice and everyday life. Collectively, these studies offer a deeper understanding of how chrononutrition should be considered to promote health and wellbeing in our modern, round-the-clock society.

Healthcare professionals should not only consider the types and quantities of food individuals consume but also the timing of meals as a crucial aspect of personalized health strategies (6). Evaluating chrononutrition involves a multifaceted approach that includes behaviors such as fasting duration, the number of main meals, meal skipping, and the timing of breakfast and dinner, alongside genetic variability (7, 8). Furthermore, research has revealed the circadian clock's role in regulating the differentiation of adipose tissues, highlighting the influence of clock proteins and their associated signaling pathways on the function and differentiation of white and brown adipose tissues (9).

Addressing chronodisruption—defined as the misalignment of biological circadian rhythms due to irregular sleep patterns, eating habits, or shift work—is essential in clinical practice to effectively prevent and manage diet-related chronic diseases. Chronodisruption has been linked to various health issues, including metabolic syndrome, obesity, diabetes, cardiovascular diseases, and mental health disorders (5). By incorporating considerations of chronodisruption into clinical practice, healthcare providers can implement interventions that align meal timing with an individual's circadian rhythm, leading to improved outcomes for both metabolic and mental health (10).

Moreover, personalized chrononutrition strategies and chronomedicine (11) present promising avenues for the prevention and treatment of chronic health conditions, ultimately enhancing patient care and overall wellbeing (12).
